# Roles of Bulk and Surface Thermodynamics in the Selective Adsorption of a Confined Azeotropic Mixture

**DOI:** 10.1021/acs.jpcb.6c00640

**Published:** 2026-04-09

**Authors:** Katie L. Y. Zhou, Anna T. Bui, Stephen J. Cox

**Affiliations:** † Department of Chemistry, 3057 Durham University, South Road, Durham DH1 3LE, United Kingdom; ‡ Yusuf Hamied Department of Chemistry, 2152 University of Cambridge, Lensfield Road, Cambridge CB2 1EW, United Kingdom

## Abstract

Fluid mixtures that exhibit an azeotrope cannot be purified by simple bulk distillation. Consequently, there is a strong motivation to understand the behavior of azeotropic mixtures under confinement. We address this problem using a machine-learning-enhanced classical density functional theory (cDFT) applied to a binary Lennard–Jones mixture that exhibits azeotropic phase behavior. As proof-of-principle of a “train once, learn many” strategy, our approach combines a neural functional trained on a single-component repulsive reference system with a mean-field treatment of attractive interactions, inspired by the connection between cDFT and local molecular field theory (LMFT). The theory faithfully describes capillary condensation and results from grand canonical Monte Carlo simulations. Moreover, by taking advantage of a known accurate equation of state, the “neural LMFT” we present well-describes bulk thermodynamics by construction. Exploiting the computational efficiency of neural LMFT, we systematically evaluate adsorption selectivity across a wide range of compositions, pressures, temperatures, and wall–fluid affinities. In cases where the wall–fluid interaction is the same for both species, we find that the pore becomes completely unselective at the bulk azeotropic composition. Strikingly, this unselective point persists far from liquid–vapor coexistence, including in the supercritical regime. Analysis of the bulk equation of state across a wide range of thermodynamic state points shows that the azeotropic composition coincides with equal partial molar volumes and an extremum in the isothermal compressibility. A complementary thermodynamic analysis demonstrates that unselective adsorption corresponds to an aneotrope (a point of zero relative adsorption) and an extremum in the interfacial free energy. We also find that the two interfaces of the slit pore behave independently down to remarkably small slits.

## Graphical abstract

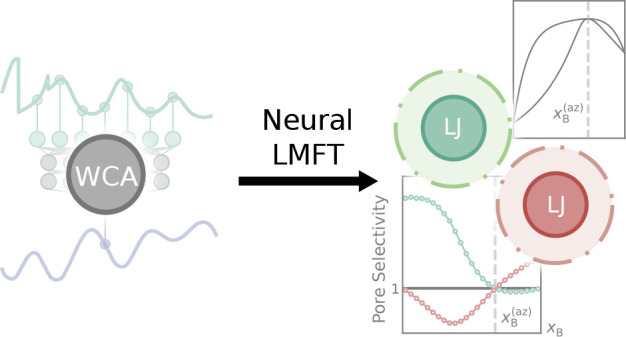

## 1 Introduction

A detailed understanding of the thermophysical properties of fluid mixtures under confinement is crucial for a wide range of applications, including industrial separation processes, gas storage, and healthcare.^1–3^ Specific examples span oil recovery,^4^ hydrogen storage,^5,6^ and microfluidic systems.^7,8^ From a theoretical standpoint, molecular simulations are widely regarded as the method of choice,^9–14^ offering microscopic insight that is often inaccessible to experiment alone.^15,16^ However, traditional approaches rooted in liquid state theory are experiencing a renaissance.^17,18^ In particular, classical density functional theory (cDFT) is attracting renewed interest in physical chemistry, driven by the ability of machine learning (ML) to deliver highly accurate and computationally efficient approximations.^19–27^

In this article, our aim is to demonstrate a recently proposed ML-based approach to cDFT for a simple Lennard–Jones (LJ) fluid mixture. While similar ideas have been explored recently in ref 21, our approach differs in two key respects. First, methodologically, we train the ML model on a repulsive *single-component* reference system and incorporate attractive interactions through a simple mean-field treatment—an approach that enhances transferability among systems. Second, from an application standpoint, we focus on a binary mixture with asymmetric interactions, which exhibits azeotropic phase behavior. As we will see, the existence of an azeotrope appears to strongly influence the fluid’s behavior—both in bulk and under confinement—across a broad range of thermodynamic conditions.

The potential appeal of cDFT over molecular simulations becomes immediately evident when considering its fundamental formalism.^28,29^ The central object in cDFT is the grand potential functional

$$\Omega_{V}([\{\varrho_{\alpha }\}],T)=F_{\mathrm{intr}}^{\left(\mathrm{id}\right)}([\{\varrho_{\alpha }\}],T)+F_{\mathrm{intr}}^{\left(\mathrm{ex}\right)}([\{\varrho_{\alpha }\}],T)+\sum_{\alpha }\int dr\varrho_{\alpha }(r)[V_{\alpha }(r)-\mu_{\alpha }]$$ 1

where $F_{\mathrm{intr}}^{\left(\mathrm{id}\right)}$ and $F_{\mathrm{intr}}^{\left(\mathrm{ex}\right)}$ are, respectively, the ideal and excess intrinsic Helmholtz free energy functionals, and *T* is the temperature. A particle of species α, with chemical potential μ_α_, experiences an external one-body potential *V*_α_, while ϱ_α_ denotes its average one-body density, though not necessarily at equilibrium. Functional minimization of Ω_*V*_ with respect to ϱ_α_ yields the equilibrium one-body density of species α, ρ_α_, which satisfies the Euler–Lagrange equation,

$$\Lambda_{\alpha }^{3}\rho_{\alpha }\left(r\right)=\exp\left(-\beta \left[V_{\alpha }\left(r\right)-\mu_{\alpha }\right]+c_{\alpha }^{\left(1\right)}\left(r;\;\left[\left\{\rho_{\alpha }\right\}\right],T\right)\right)$$ 2

with Λ_α_ denoting the thermal de Broglie wavelength of species α, β = 1/*k*_B_ *T* (*k*_B_ is the Boltzmann constant), and

$$c_{\alpha }^{\left(1\right)}(r;\;[\{\varrho_{\alpha }\}],T)=-\frac{\delta \beta F_{\mathrm{intr}}^{\left(\mathrm{ex}\right)}([\{\varrho_{\alpha }\}],T)}{\delta \varrho_{\alpha }(r)}$$ 3

is the one-body direct correlation functional. In the context of the Euler–Lagrange equation, *c*_α_^(1)^ acts as a self-consistent one-body potential that accounts for the effects of correlations on the structure of the fluid. With the equilibrium densities from eq 2, the grand potential of the system readily follows: Ω = Ω_*V*_([{ρ_α_}], *T*). The procedure for obtaining the structure and thermodynamics of the system can, therefore, be succinctly put: minimize a functional to obtain the equilibrium structure, then evaluate the functional at equilibrium to obtain the thermodynamic potential of the system. This is a far simpler operation than explicitly sampling the many-body equilibrium distribution function with molecular simulations.

What, then, has prevented cDFT from becoming the method of choice for understanding the equilibrium properties of fluids? The answer is straightforward. While a rigorous theoretical framework, in practice cDFT relies upon approximations to $F_{\mathrm{intr}}^{\left(\mathrm{ex}\right)}$ . (Only for hard rods in a single dimension is $F_{\mathrm{intr}}^{\left(\mathrm{ex}\right)}$ known exactly.^30^) In the case of hard spheres, very good approximations founded on Rosenfeld’s fundamental measure theory (FMT) have been available for several decades.^31–34^ When combined with an *ad hoc* mean-field treatment of attractive interactions, cDFT has been shown to be a powerful practical method for understanding the physics of simple liquids. In particular, the physics of capillarity,^35,36^ surface drying and wetting,^37,38^ and solvophobicity^39^ have been extensively studied in single-component systems, owing to the ability of the mean-field approximation to qualitatively capture liquid–gas phase coexistence. While less intensely studied compared to single-component fluids, such a mean-field approach has also provided qualitative insight into the behavior of simple mixtures,^40–47^ though the richer phase behavior of multicomponent fluids—including liquid–liquid coexistence, azeotropy and heteroazeotropy^48^—pushes the limits of analytical approximations for $F_{\mathrm{intr}}^{\left(\mathrm{ex}\right)}$ .

Going beyond hard spheres and a mean-field treatment for attractive interactions is challenging, though we note significant progress with analytical approaches has been made.^49–52^ Naturally, several groups have recently turned to ML. For example, early works by Oettel and co-workers focused on one-dimensional systems^27,53^ and anisotropic patchy particles,^54^ while Cats et al. used ML to improve the standard mean-field approximation for the three-dimensional LJ fluid.^25^ One ML scheme that is gaining significant traction is “neural functional theory”^19^ introduced by Sammüller et al. The basis of this physics-informed approach is the Euler–Lagrange equation (eq 2). For known {*V*_α_} and {μ_α_}, with {ρ_α_} obtained from grand canonical Monte Carlo (GCMC) simulation, the spatial variation of each *c*_α_^(1)^ at equilibrium is determined by rearranging eq 2:

$$c_{\alpha }^{\left(1\right)}(r;\;[\{\rho_{\alpha }\}],T)=\ln\;\Lambda_{\alpha }^{3}\rho_{\alpha }(r)+\beta (V_{\alpha }(r)-\mu_{\alpha })$$ 4

By obtaining {*c*_α_^(1)^} from many GCMC simulations with different {*V*_α_}, {μ_α_}, and equilibrium density profiles {ρ_α_}, a training set is established to learn the local functional dependence of *c*_α_^(1)^ on {ϱ_α_} with a neural network.

Originally developed for the single-component hard-sphere fluid—where it even outperformed FMT-based functionals^19^—the neural cDFT approach has since been extended to more complex systems. For example, Sammüller et al. applied neural cDFT to the LJ fluid, demonstrating accurate predictions of liquid–vapor coexistence.^22^ Building on this, Robitschko et al. showed that liquid–liquid coexistence in a simple binary LJ mixture is also well captured.^21^ By incorporating orientational correlations, Yang et al. have successfully applied neural cDFT to molecular CO_2_.^26^

While neural cDFT exploits the local nature of correlations, Bui and Cox addressed systems with long-ranged electrostatic interactions,^20^ combining neural cDFT with a systematically improvable mean-field approach inspired by local molecular field theory (LMFT).^55^ Initially developed for primitive electrolyte models,^20^ this LMFT-style framework was later generalized to systems with nontrivial coupling between charge and number density, such as polar fluids.^23,56^ At the foundation of this generalization lies hyperdensity functional theory^57^ (hyper-DFT), also introduced by Sammüller et al. in the context of soft matter, which establishes that any equilibrium observable can be expressed as a functional of the one-body density. Leveraging hyper-DFT, Bui and Cox established a rigorous framework for electromechanics in fluids^56^ and demonstrated that electric field gradients can alter liquid–vapor coexistence in polar fluids, including water.^23^ This work uncovered a previously unknown effect—dielectrocapillarity—in which field gradients control adsorption into porous media.

Clearly, the neural functional approach offers a route to fully harness the advantages of cDFT over molecular simulations. While the LMFT-based approach arguably remains essential for systems dominated by long-range interactions, many systems can now be tackled simply by learning {*c*_α_^(1)^}, without invoking any of the standard approximations of liquid state theory. In this light, can traditional strategies—such as decomposing the system into a repulsive reference plus mean-field attraction, as in FMT-based approaches—still play a meaningful role?

We argue that the answer is “yes.” Our reasons are twofold. First, when the bulk equation of state (EoS) is well established by other means, the LMFT-based framework described in ref 56 offers a straightforward way to leverage the rich information that it encodes. By adopting this strategy, we can focus efforts where they are most needed: neural cDFT can be targeted to the study of inhomogeneous systems where its advantages are most pronounced, leaving the bulk physics to the already-known EoS. Second, while cDFT itself is more efficient than molecular simulations, neural cDFT comes with an initial computational overhead in obtaining the training data. Decomposing the system into a repulsive reference—treated with neural cDFT—and mean-field attractive interactions, opens the door to a “train once, learn many” strategy, where a single, accurate repulsive reference can be reused across multiple systems. Such an approach becomes particularly advantageous in the case of mixtures; in this article, we demonstrate a proof-of-principle for a binary LJ mixture with asymmetric interactions that exhibits azeotropy.

The rest of the article is organized as follows. In Section 2.1, we introduce the LJ mixture and outline the underpinning LMFT-based framework. Sections 2.2–2.4 provide details on the neural cDFT setup. In Sections 3.1 and 3.2, we validate the approach against GCMC simulations and examine adsorption and selectivity in a slit-pore that interacts with both species in the same manner. We analyze the bulk thermodynamic behavior of the mixture, with particular emphasis on the role of the azeotropic composition across a wide range of pressures and temperatures, in Section 3.3. In Section 3.4, we develop a thermodynamic description of pore selectivity that sheds light on the underlying driving forces for selective adsorption. Finally, Section 4 summarizes our main findings and discusses their broader implications and possible extensions.

## 2 Methods

### 2.1 Neural LMFT

The intermolecular interactions that govern fluid mixtures can, in general, be complicated, potentially comprising strongly directional interactions in the form of hydrogen bonds,^58^ long-ranged electrostatic effects,^59,60^ and even metallicity.^61^ Nonetheless, many of the most salient aspects of fluid mixtures are captured by relatively simple forms for the intermolecular interactions. For instance, complex liquid–vapor and liquid–liquid phase equilibria are reproduced with LJ mixtures,^62,63^ alkane phase separation with generalized Mie potentials,^64^ binary alloys with Stillinger–Weber^65,66^ and Tersoff^67,68^ potentials, and polymer demixing with Gaussian core models.^69^

Here, the system we investigate is a binary mixture of species A and B, whose pairwise interactions are prescribed by the truncated and shifted LJ pair potential,

$$u_{\alpha \eta }\left(r\right)=4\epsilon_{\alpha \eta }\left[{\left(\sigma /r\right)}^{12}-{\left(\sigma /r\right)}^{6}\right]-4\epsilon_{\alpha \eta }\left[{\left(\sigma /r_{c}\right)}^{12}-{\left(\sigma /r_{c}\right)}^{6}\right]$$ 5

for *r* < *r*_c_ (and zero otherwise), where α and η are species labels, *r*_c_ = 2.5σ, and the molecular diameter, σ, is the same for all particles. In contrast, the strength of interaction between particles is species-dependent. Specifically, ϵ_AA_ = ϵ, ϵ_BB_ = 0.9ϵ, and ϵ_AB_ = 0.806ϵ. In Figure 1a we present the predicted bulk phase diagram from the PeTS EoS^70,71^ at a temperature *k*_B_ *T*/ϵ = 0.77, in the *P*–*x*_B_ plane, where *P* is pressure and *x*_B_ is the mole fraction of B. Previous studies^62,63,72^ have shown that, at this temperature, this system exhibits a positive azeotrope—the point on the phase diagram at which liquid and vapor have the same composition—at mole fraction *x*_B_^(az)^ ≈ 0.67 and pressure *P* ^(az)^σ^3^/ϵ = 0.0248.

**Figure 1:**
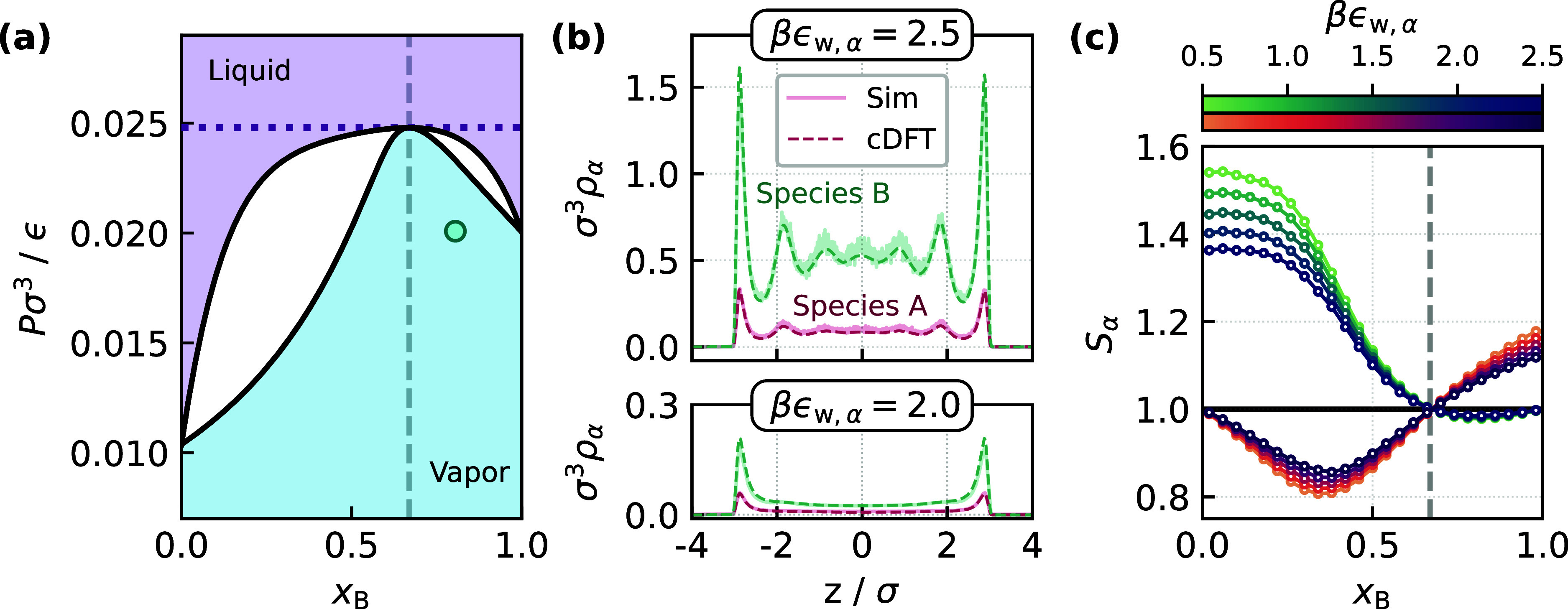
Effects of confinement on a binary LJ mixture. (a) The pressure–composition phase diagram of the mixture at *k*_B_ *T*/ϵ = 0.77. An azeotrope forms at *x*_B_^(az)^ ≈ 0.67, as indicated by the vertical dashed line. (b) Density profiles for the mixture confined in a slit pore, in equilibrium with a reservoir at *P*σ^3^/ϵ = 0.020, and *x*_B_ = 0.78 [indicated by the circle in (a)]; we consider two different wall–fluid interaction strengths, as indicated by the labels. Good agreement between the theory and the simulation data is observed. (c) Pore selectivities vs *x*_B_, at the azeotropic pressure [indicated by the dotted line in (a)], for different wall–fluid interactions. Results are shown for both component A (orange to purple) and B (green to blue). Lines serve as a guide to the eye. A reversal in selectivity is observed at *x*_B_^(az)^, as indicated by the vertical dashed line.

To describe the structure and thermodynamics of this system in a density functional framework, we will adopt an LMFT-based approach to neural cDFT, which we refer to as “neural LMFT” for convenience. In LMFT, as developed by Weeks and co-workers,^73–75^ a short-ranged reference system with pairwise interactions *u*_0,αη_(*r*) is introduced. For this reference system, we then seek the set of one-body potentials {ϕ_R,α_} such that the resulting equilibrium one-body densities satisfy ρ_R,α_(***r***) = ρ_α_(***r***) for all α, where the subscript “R” indicates properties pertaining to the reference system.

The formal similarity between LMFT and cDFT in a mean-field approximation was originally established by Archer and Evans,^76^ though the well-controlled nature of the mean-field form that underlies LMFT was left implicit.^75^ Building on ref 76, LMFT’s equivalence to hyper-DFT was also recently formalized,^56^ re-establishing the well-controlled nature of the mean-field approximation.

More important for the present study, however, is that ref 56 asserted a mean-field free energy functional in which the bulk and inhomogeneous contributions are cleanly separated,

$$F_{\mathrm{intr}}^{\left(\mathrm{ex}\right)}([\{\varrho_{\alpha }\}],T)=F_{\mathrm{intr},R}^{\left(\mathrm{ex}\right)}([\{\varrho_{\alpha }\}],T)+\sum_{\alpha }\Delta \mu_{\alpha }\int dr\varrho_{\alpha }(r)+\frac{1}{2}\sum_{\alpha ,\eta }\int dr\int dr^{\prime }\delta_{u}\varrho_{\alpha }(r)u_{1,\alpha \eta }(|r-r^{\prime }|)\delta_{u}\varrho_{\eta }(r^{\prime })$$ 6

where Δμ_α_ = μ_α_ – μ_R,α_ is the difference in chemical potentials between the LJ and reference systems, *u*_1,αη_ = *u*_αη_ – *u*_0,αη_, and δ_u_ϱ_α_ = ϱ_α_ – ρ̅_α_, with ρ̅_α_ denoting the uniform bulk density for species α for given {μ_α_} and *T*.

With such a mean-field form, following either ref 56 or ref 76, one can show that

$$c_{\alpha }^{\left(1\right)}(r;\;[\{\rho_{\alpha }\}],T)=c_{R,\alpha }^{\left(1\right)}(r;\;[\{\rho_{\alpha }\}],T)-\beta \phi_{R,\alpha }(r)-\beta \Delta \mu_{\alpha }$$ 7

where

$$\phi_{R,\alpha }\left(r\right)=\underset{\eta }{\sum }\int dr^{\prime }u_{1,\alpha \eta }\left(|r-r^{\prime }|\right)\delta_{u}\rho_{\eta }\left(r^{\prime }\right)$$ 8

The separation of bulk and inhomogeneous contributions to the mean-field free energy functional affords a level of flexibility that can be put to practical advantage; an accurate description of the bulk fluid can be obtained if {Δμ_α_} is known by any means. For example, in the case of primitive models of electrolytes and dielectric fluids, analytic expressions for {Δμ_α_} have been derived based on Stillinger–Lovett sum rules.^20,56^ Here, we will take advantage of the fact that {Δμ_α_} can be obtained from a known EoS.

Success of this neural LMFT approach rests upon a suitable choice of reference system. For the binary LJ mixture that we study here, in which A and B have the same molecular diameter, we choose the reference system to be the purely repulsive *single-component* fluid described by the Weeks–Chandler–Anderson (WCA) potential,^77^ i.e., *u*_0,αη_ = *u*_0_, with

$$u_{0}\left(r\right)=\{\begin{array}{ll} 4\epsilon \left[{\left(\sigma /r\right)}^{12}-{\left(\sigma /r\right)}^{6}\right]+\epsilon , & r<2^{1/6}\sigma \\ 0, & r\geq 2^{1/6}\sigma \end{array}$$ 9

Combining eqs 2 and 7, the Euler–Lagrange equation for species α of the LJ mixture reads,

$$\Lambda_{\alpha }^{3}\rho_{\alpha }(r)=\exp(-\beta [V_{\alpha }(r)+\phi_{R,\alpha }(r)-\mu_{R,\alpha }]+c_{R}^{\left(1\right)}(r;\;[\rho_{A}+\rho_{B}],T))$$ 10

with

$$\mu_{R,\alpha }({\overset{-}{\rho }}_{A},{\overset{-}{\rho }}_{B})=k_{B}T\;\ln\;\Lambda_{\alpha }^{3}{\overset{-}{\rho }}_{\alpha }-k_{B}Tc_{R}^{\left(1\right)}([{\overset{-}{\rho }}_{A}+{\overset{-}{\rho }}_{B}],T)$$ 11

### 2.2 Generation of Training Data

Following ref 19, we trained a neural functional to represent *c*_R_^(1)^ of the WCA fluid, using data from 900 GCMC simulations. In practice, the neural functional is limited to a planar geometry, i.e., *c*_R_^(1)^(***r***; [{ϱ_α_}], *T*) → *c*_R_^(1)^(*z*; [{ϱ_α_}], *T*); we direct the reader toward ref 78 for recent developments that extend the neural functional approach to resolution in higher dimensions.

Simulations were performed using our own code available on Github (https://github.com/annatbui/gcmc) or Zenodo.^79^ They were conducted at temperatures *k*_B_ *T*/ϵ = 1.0, 1.5, and 2.0 with randomized chemical and external potentials. The chemical potentials were chosen from the range −10 ≤ βμ ≤ 3 and the external potentials had the form

$$V\left(z\right)=\overset{4}{\underset{n=1}{\sum }}A_{n}\sin\left(\frac{2\pi nz}{l}+\Phi_{n}\right)+\underset{n}{\sum }V_{n}^{\mathrm{lin}}\left(z\right)$$ 12

where *A*_*n*_ were randomly chosen Fourier coefficients from a normal distribution of variance 2.5­(*k*_B_ *T*)^2^ and the phases Φ_*n*_ were chosen uniformly between 0 and 2π. The simulation box was cubic with length l=10σ and periodic boundary conditions were applied. The linear function *V*_*n*_^lin^(*z*) takes the form

$$V_{n}^{\mathrm{lin}}\left(z\right)=V_{n,1}+\frac{\left(V_{n,2}-V_{n,1}\right)\left(z-z_{n,1}\right)}{\left(z_{n,2}-z_{n,1}\right)}$$ 13

for *z*_*n*,1_ < *z* < *z*_*n*,2_ and 0 otherwise, where *z*_*n*,1_ and *z*_*n*,2_ were uniformly chosen such that $0<z_{n,1}<z_{n,2}<l$ , and *V*_*n*,1_ and *V*_*n*,2_ were randomly chosen from a normal distribution with variance 4­(*k*_B_ *T*)^2^. Each external potential had four sinusoidal segments, and between 1 and 5 linear segments. Half of the potentials had planar hard walls, where *V*(*z*) = ∞ for *z* ≤ *z*_w_/2 and $z\geq l-z_{w}/2$ ; *z*_w_ was randomly chosen uniformly between 1σ and 3σ.

Each simulation was run for 10^9^ steps, with equilibration for 10^6^ steps. For each simulation, the planar density profile ρ­(*z*) was obtained from a histogram of the positions of the particles, and *c*_R_^(1)^(*z*; [ρ], *T*) was obtained from numerical evaluation of the single-component version of eq 4. The total computation time for the generation of the entire training data set is on the order of 10^4^ CPU hours.

### 2.3 Training the Neural Functional

Our training procedure largely follows that of previous work.^19–22^ A neural network was trained to represent *c*_R_^(1)^(*z*; [ρ], *T*), implemented using Keras/Tensorflow with the standard Adam optimizer.^80^ The input layers take in the temperature and the density in a window of size 3σ around the location of interest, with spatial discretization Δ*z* = 0.005σ. This was followed by two hidden layers of 32 nodes with softplus activation functions, and then the single-node output. The simulation data set was split 3:1:1 for the training, validation, and test data sets, respectively. Data augmentation allowed the doubling of the training data through mirroring. The model was trained for 100 epochs in batches of size 256 with the mean squared error as the loss function. The initial learning rate was 0.001, decreasing by 5% per epoch. The training was done on an NVIDIA GH200 Grace Hopper Superchip in under an hour; we stress that use of this architecture was made out of convenience due to available resources, and training is entirely possible on commodity hardware.^19–23^

### 2.4 Using the Neural Functional

Once the neural functional has been trained, combining with LMFT using eq 7 gives *c*_α_^(1)^(*z*; [{ρ_α_}], *T*). Given a bulk state point, μ_α_(ρ̅_A_, ρ̅_B_) can be obtained directly from the PeTS EoS^70^ (here we use its implementation from the FeOs package^71^ version 0.8.0); Δμ_α_ is then obtained by subtracting μ_R,α_(ρ̅_A_, ρ̅_B_) given by eq 11. Minimization of the Euler–Lagrange equation to obtain density profiles is done self-consistently using a mixed Picard scheme, and typically takes around 2 min on a standard CPU (and faster on a GPU).

To evaluate the grand potential of the binary LJ mixture, we use eq 1 with $F_{\mathrm{intr}}^{\left(\mathrm{ex}\right)}$ obtained by functional line integration,^19^

$$\beta F_{\mathrm{intr}}^{\left(\mathrm{ex}\right)}/A=-\int_{0}^{1}d\lambda \sum_{\alpha }\int dz\rho_{\alpha }(z)c_{\alpha }^{\left(1\right)}(z;\;[\{\lambda \rho_{\alpha }\}],T)$$ 14

where A is the cross-sectional area of the slit pore, and $\beta F_{\mathrm{intr}}^{\left(\mathrm{id}\right)}/A=\underset{\alpha }{\sum }\int dz\rho_{\alpha }\left(z\right)\left(\ln\left[\Lambda_{\alpha }^{3}\rho_{\alpha }\left(z\right)\right]-1\right)$ . Overall we have performed approximately 6200 cDFT calculations, from which we directly obtain both equilibrium density profiles and free energies.

## 3 Results and Discussion

### 3.1 Neural LMFT Successfully Describes an Asymmetric Binary LJ Mixture

In Figure 1b we show results from neural LMFT for the binary LJ mixture in a slit-pore at equilibrium with a reservoir at *k*_B_ *T*/ϵ = 0.77, *P*σ^3^/ϵ = 0.020, and *x*_B_ = 0.78, which corresponds to the vapor state. In these calculations, the left and right walls of the slit-pore each act as an external potential confining the particles to a region −*L*/2 < *z* < *L*/2

$$V_{\alpha }^{\left(\mathrm{single}\right)}\left(z\right)=4\epsilon_{w,\alpha }\left[{\left(\sigma /\left(z-z_{w}\right)\right)}^{12}-{\left(\sigma /\left(z-z_{w}\right)\right)}^{6}\right]$$ 15

where *z*_w_ = ± *L*/2. Similar to the interatomic potential, each *V*_α_^(single)^ is truncated and shifted at a cutoff *z*_c_ = 2.5σ. In Figure 1b, we have considered the symmetric case, ϵ_w,*A*_ = ϵ_w,*B*_ = 2.0 *k*_B_ *T*. For a mildly attractive interaction between the confining walls and the particles, equilibrium densities predicted from the neural LMFT calculations are in excellent agreement with GCMC simulations, and overall consistent with a vapor-like state in the pore. Upon increasing the interaction strength to 2.5 *k*_B_ *T*, neural LMFT captures the transition to a liquid-like state predicted by GCMC simulations. While some minor discrepancies in the density profiles are observed for these more attractive walls, agreement between theory and simulation remains very good.

Overall, the combination of the accurate PeTS EoS for bulk, and the neural functional for the inhomogeneous correlations means that the neural LMFT approach outperforms the standard mean-field cDFT treatment across a broad range of thermodynamic conditions (see Figures S1–S3). Moreover, we also demonstrate the robustness of our approach on other LJ binary mixtures with different attractive interactions (we investigate systems with $0.85\leq \epsilon_{\mathrm{AB}}/\sqrt{\epsilon_{\mathrm{AA}}\epsilon_{\mathrm{BB}}}\leq 1.25$ ), illustrating the potential power of the “train once, learn many” strategy (see Figure S4). Clearly, success of this approach rests upon the suitability of the reference system, and an accurate EoS for the binary fluid. For our current purposes, the single-component WCA fluid is reasonable for mixtures with components of equal size, and where cross-interactions are not too dissimilar, while the PeTS EoS appears sufficiently accurate.

### 3.2 Efficient Evaluation of Pore Selectivity Unveils a Significance of the Azeotropic Composition under Confinement

Having established the accuracy of the neural LMFT approach, we now capitalize on its advantages as a density functional framework. Specifically, we investigate how confinement influences the overall composition of the fluid across a broad range of wall–fluid interaction strengths and thermodynamic conditions; this is especially important for systems that exhibit azeotropy, such as the binary LJ mixture under investigation here. While thermodynamic modeling of bulk mixtures is well-established,^82^ major gaps remain in our understanding of azeotropy under confinement (“adsorption azeotropy” ^83–86^ )—addressing this issue is of broad relevance to chemical separation and industrial processes.^87,88^

To assess the influence of confinement on the composition of the fluid, we use neural LMFT to compute the pore selectivity of each species,^11,44,45,89^

$$S_{\alpha }=\frac{N_{\alpha }/\left(N_{A}+N_{B}\right)}{x_{\alpha }}$$ 16

where *N*_α_ is the total number of adsorbed particles of species α,

$$N_{\alpha }=A\int_{-L/2}^{+L/2}dz\rho_{\alpha }\left(z\right)$$ 17

Note that *x*_α_ is a property of the bulk reservoir. In the first instance, we evaluate *S*_α_ at fixed *T* and *P* over a wide range of ϵ_w,A_ = ϵ_w,B_ and *x*_B_, as shown in Figure 1c.

Our calculations of *S*_α_ reveal that, relative to the bulk composition, species B is preferentially adsorbed at low *x*_B_, whereas species A is preferred at high *x*_B_. This observation is consistent with previous studies that have found selective adsorption for the component with weaker fluid–fluid interactions when it is the minority component in bulk.^43,90^ The most striking observation from Figure 1c, however, is that the crossover between B- and A-selectivity, where *S*_A_ = *S*_B_ = 1, occurs very close to the azeotropic composition, irrespective of the wall–fluid interaction strength; for reasons that will become clear in Section 3.4, we refer to this composition, *x* ^(an)^, as the “aneotropic composition”.^91–93^

The fact that *x*_B_^(an)^ ≈ *x*_B_^(az)^ appears insensitive to the strength of the wall–fluid interaction motivates us to investigate the extent to which the azeotropic composition influences pore selectivity under different thermodynamic conditions. To this end, we repeat our calculations at the same temperature *k*_B_ *T*/ϵ = 0.77 but at both higher and lower pressures, and again at *k*_B_ *T*/ϵ = 1.50, with the results shown in Figure 2a. The state points considered encompass regions both below and above the azeotropic and critical lines of the mixture on the *P*–*T* phase diagram, as marked in Figure 2b. Remarkably, all show the same crossover at *x*_B_^(an)^ ≈ *x*_B_^(az)^, despite being far from the azeotropic line.

**Figure 2:**
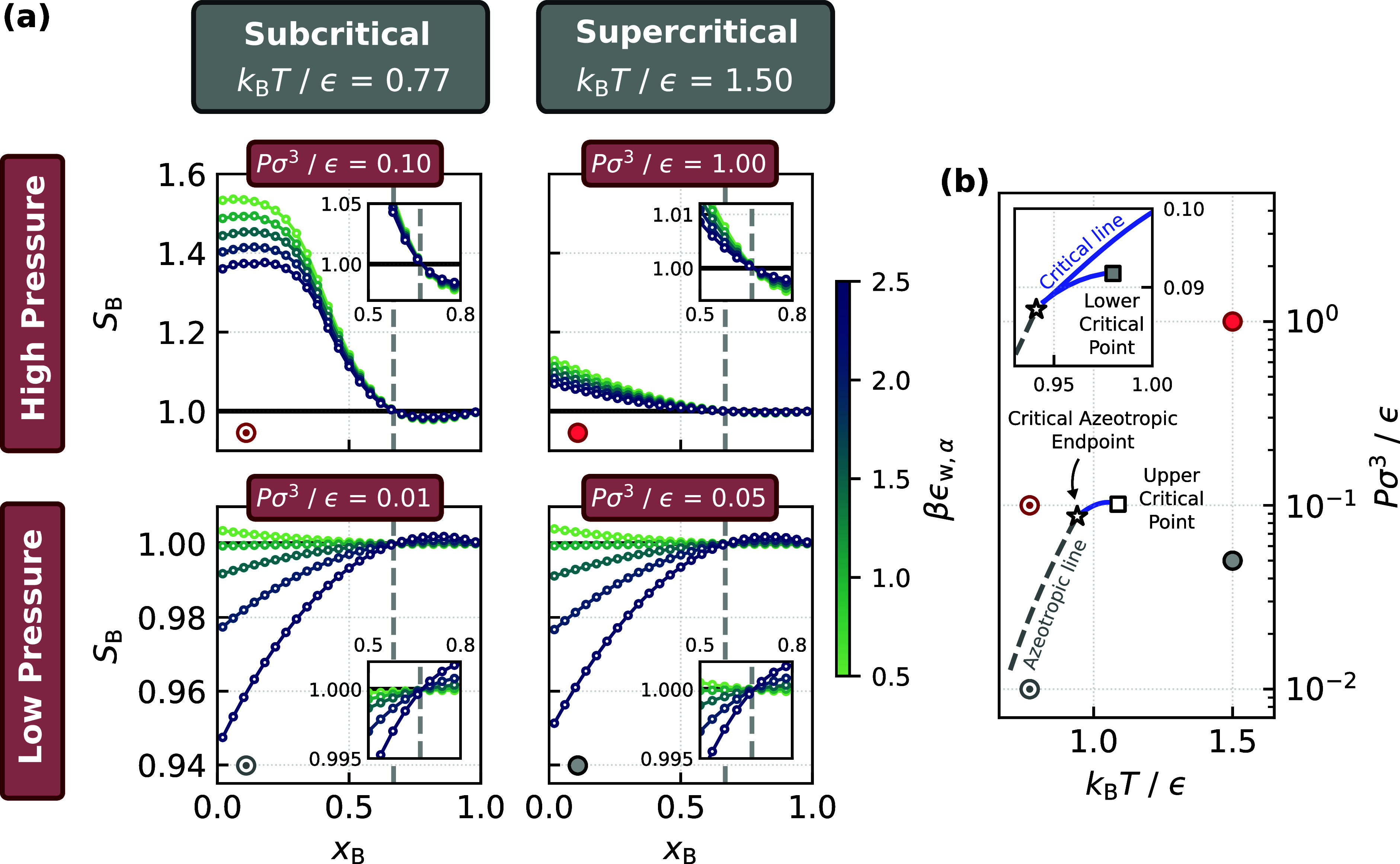
Relevance of the azeotropic composition across a broad range of thermodynamic conditions. (a) *S*_B_ vs *x*_B_ with varying wall–fluid interaction strengths (indicated by the color bar) at different state points (indicated by the labels). In all cases, a crossover in selectivity occurs at *x*_B_ ≈ *x*_B_^(az)^. (b) *P*–*T* phase diagram obtained from the PeTS EoS. The critical azeotropic end point is *k*_B_ *T*_CAEP_/ϵ ≈ 0.94 ^81^ (star) and the upper critical point is *k*_B_ *T*_C_/ϵ ≈ 1.09 (white square). The inset shows the region around *k*_B_ *T*_CAEP_/ϵ with the lower critical point. The circles indicate the thermodynamic conditions used in (a).

So far, we have investigated slit pores that interact with species A and B in an identical fashion. Yet, even in this simple symmetric case, we observe selective adsorption. Moreover, we observe completely unselective behavior close to the azeotropic composition of the *bulk* fluid. Before going on to investigate how pore selectivity may vary in the case of asymmetric wall–fluid interactions, we therefore aim to understand the behavior of the bulk fluid in greater detail.

### 3.3 Bulk Thermodynamics and the Robust Signature of Azeotropy

The results we present in Figures 1c and 2a indicate that the azeotropic composition remains relevant to the mixture’s adsorption behavior even at thermodynamic conditions far from liquid–vapor coexistence. To shed light on these observations, we recall that the azeotrope is defined as the point of liquid–vapor coexistence at which the composition of the two phases in bulk is the same: *x*_B_^(l)^ = *x*_B_^(v)^ = *x*_B_^(az)^, where the superscripts “(l)” and “(v)” indicate quantities that pertain to the liquid and vapor phases, respectively. Therefore, in addition to mechanical and thermal equilibrium, we have^48^

$$\mu_{B}^{\left(l\right)}\left(x_{B}^{\left(\mathrm{az}\right)},P^{\left(\mathrm{az}\right)},T^{\left(\mathrm{az}\right)}\right)=\mu_{B}^{\left(v\right)}\left(x_{B}^{\left(\mathrm{az}\right)},P^{\left(\mathrm{az}\right)},T^{\left(\mathrm{az}\right)}\right)$$ 18a

$$\mu_{A}^{\left(l\right)}\left(x_{B}^{\left(\mathrm{az}\right)},P^{\left(\mathrm{az}\right)},T^{\left(\mathrm{az}\right)}\right)=\mu_{A}^{\left(v\right)}\left(x_{B}^{\left(\mathrm{az}\right)},P^{\left(\mathrm{az}\right)},T^{\left(\mathrm{az}\right)}\right)$$ 18b

where, *T* ^(az)^ and *P* ^(az)^ are the temperature and pressure along the azeotropic line^81^ in the *P*–*T* plane (see Figure 2b). Introducing the exchange potential,

$$\Delta_{E}\mu \left(x_{B},P,T\right)=\mu_{B}\left(x_{B},P,T\right)-\mu_{A}\left(x_{B},P,T\right)$$ 19

and subtracting eq 18b from eq 18a, we find

$$\Delta_{E}\mu^{\left(l\right)}\left(x_{B}^{\left(\mathrm{az}\right)},P^{\left(\mathrm{az}\right)},T^{\left(\mathrm{az}\right)}\right)=\Delta_{E}\mu^{\left(v\right)}\left(x_{B}^{\left(\mathrm{az}\right)},P^{\left(\mathrm{az}\right)},T^{\left(\mathrm{az}\right)}\right)$$ 20

Thus, under azeotropic conditions, the reversible work required to exchange a particle of species A for one of species B, as encoded in Δ_E_μ, is identical in the liquid and vapor phases.

Inspired by the observation that the azeotropic composition appears relevant across a broad range of *P* and *T*, we investigate the behavior of the exchange potential away from coexistence. To this end, in Figure 3a we show how Δ_E_μ varies with *x*_B_ for different *P* and *T*; note that these results are obtained directly from the PeTS EoS. At subcritical temperatures, we see that Δ_E_μ­(*x*_B_) obtained at different pressures collapse into two distinct branches, corresponding to the vapor and liquid states. Strikingly, we observe that these two branches cross at *x*_B_^(az)^. At supercritical temperatures, while no longer separated into liquid and vapor branches, the observation that Δ_E_μ­(*x*_B_^(az)^) is equal for all *P* persists. Across the temperature range we consider, these results imply that

$${\left(\frac{\partial \Delta_{E}\mu }{\partial P}\right)}_{x_{B}=x_{B}^{\left(\mathrm{az}\right)},T}=0$$ 21

An alternative, but equivalent, viewpoint is that at the azeotropic composition, irrespective of *P* and *T*, the partial molar volumes, ${\tilde{v}}_{\alpha }=(\partial \mu_{\alpha }/\partial P{)}_{x,T}$ , of A and B are identical. We stress that we have not derived eq 21; it is an observation based on the results in Figure 3a.

**Figure 3:**
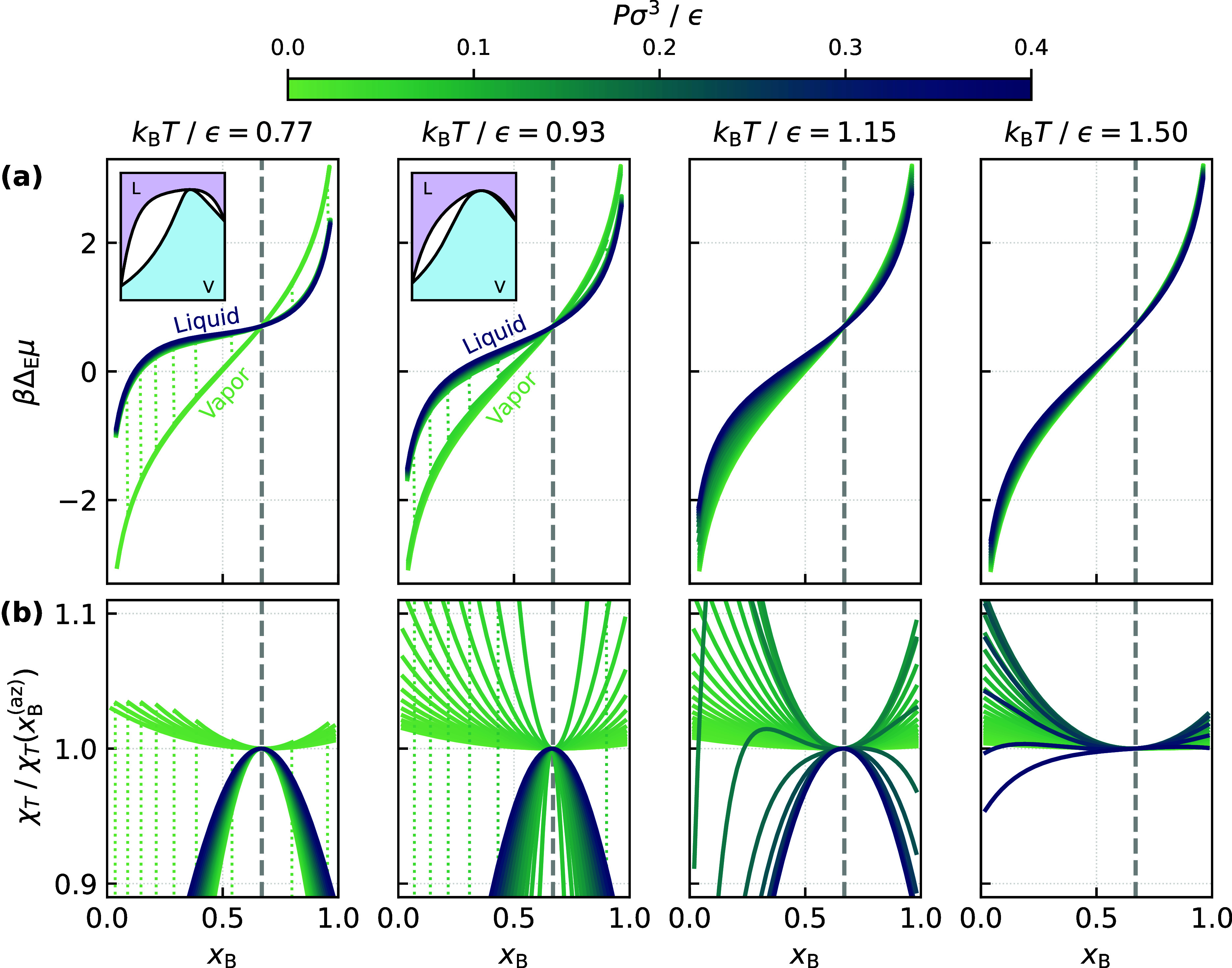
Bulk thermodynamic properties vs *x*_B_ for different *P* and *T* obtained from the PeTS EoS. From left to right, the temperatures correspond to *T* ≪ *T*_c_, *T* ≲ *T*_CAEP_ < *T*_c_, *T* > *T*_c_, and *T* ≫ *T*_c_. (a) For subcritical temperatures, Δ_E_μ collapses into liquid and vapor branches that cross at *x*_B_ ≈ *x*_B_^(az)^; this crossing point persists at supercritical temperatures. The insets show schematic representations of the *P*–*x*_B_ phase diagram at subcritical temperatures. (b) The isothermal compressibility is locally extremum at *x*_B_ ≈ *x*_B_^(az)^ at all temperatures. In both (a) and (b), discontinuities representing liquid–vapor phase transitions are shown with dotted lines.

For single-component fluids, there has been much interest in characterizing the nature of the supercritical state. In particular, while the critical point is defined by the indistinguishability of liquid and vapor states, several studies suggest regions in the phase diagram—often separated by so-called “Widom lines”—where the supercritical state behaves more liquid- or vapor-like.^94–99^ To our knowledge, the supercritical state of binary fluids remains much less intensely studied.^100,101^ In this context, the observation of identical partial molar volumes at *x*_B_^(az)^ at *T* ≫ *T*_c_ is intriguing. While an exhaustive study into this topic remains beyond the scope of this article, in Figure 3b we show how the isothermal compressibility,

$$\chi_{T}(x_{B},P,T)=\frac{1}{\mathrm{\rho ̅}}(\frac{\partial \overset{-}{\rho }}{\partial P}{)}_{x_{B},T}$$ 22

where ρ̅ = ρ̅_A_ + ρ̅_B_, varies with *x*_B_ for different *P* and *T*. Though not unique, locating conditions of maximum isothermal compressibility is a common approach to mapping Widom lines in single-component systems.^94–99^ For the binary system we consider, we see that χ_*T*_(*x*_B_^(az)^, *P*, *T*) is locally extremum. Specifically, at subcritical temperatures, we observe that vapor and liquid states are characterized by positive and negative curvatures, respectively. Remarkably, hallmarks of this observation persist at supercritical temperatures, with χ_*T*_(*x*_B_^(az)^, *P*, *T*) a local minimum at low pressures, and a local maximum at high pressures. For now, we leave this as an intriguing observation. Our initial results for pore selectivity (Figures 1c and 2a), however, hint that such supercritical behavior of the bulk fluid influences behavior under confinement.

### 3.4 Thermodynamic Origin and Control of Selective Adsorption in Confined Azeotropic Mixtures

For the pores that we have considered so far, i.e., those that interact with species A and B in the same manner, our analysis indicates that the bulk thermodynamics of the fluid mixture play an important role in determining selective adsorption under confinement. While the observations that the partial molar volumes of A and B are equal at the azeotropic composition, and *x*_B_^(an)^ ≈ *x*_B_^(az)^, are intriguing, they do not by themselves provide a clear mechanistic explanation of the observed adsorption behavior. More generally, there has been significant interest in understanding the mechanisms of selective adsorption under confinement, especially when B assumes the role of a minority species dissolved in A, e.g., carbon dioxide in water; in such cases, *S*_B_ is often dubbed “oversolubility.” In this context, there has been much work discussing the roles of adsorption vs confinement-induced changes on the solubility (see, e.g., ref 89 for a review). Here, we present a simple thermodynamic analysis that sheds light on the driving forces that underpin *S*_B_. Anticipating the results that follow, we find that, in the thermodynamic sense, *S*_B_ is driven solely by adsorption.

Following standard treatments of the thermodynamics of confined fluids,^29,36^ our starting point is the exact differential of the surface excess grand potential,

$$d\Omega^{\left(\mathrm{ex}\right)}=-2sAdT-A\underset{\alpha }{\sum }\Gamma_{\alpha }d\mu_{\alpha }+2\gamma dA-f_{s}AdH$$ 23

where *s* is excess entropy per unit area, *f*_s_ is the solvation force, γ is the wall–fluid interfacial tension,

$$\gamma =\frac{\Omega +PV}{2A}$$ 24

where V=AH and *P* the bulk pressure, and

$$\Gamma_{\alpha }=\int_{-H/2}^{H/2}dz(\rho_{\alpha }(z)-{\overset{-}{\rho }}_{\alpha })=N_{\alpha }/A-{\overset{-}{\rho }}_{\alpha }H$$ 25

is the adsorption of species α. Note that, in this thermodynamic picture, there is a degree of flexibility in choosing *H*, the separation between the two walls; it need not be the same as the separation *L* that is used in the external potential (see eq 15). To be consistent with *N*_α_ used in eq 16, however, *H* should be large enough to encompass all particles in the slit pore (we discuss below how we choose *H* in practice). As $\Omega^{\left(\mathrm{ex}\right)}=2\gamma A$ , from eq 23 one can straightforwardly obtain the Gibbs adsorption equation for mixtures,

$$2d\gamma +2sdT+\underset{\alpha }{\sum }\Gamma_{\alpha }d\mu_{\alpha }+f_{s}dH=0$$ 26

from which it immediately follows that

$$\Gamma_{\alpha }=-2{\left(\partial \gamma /\partial \mu_{\alpha }\right)}_{\mu_{\eta \neq \alpha },H,T}$$ 27

From the definitions of *S*_α_ (eq 16) and Γ_α_ (eq 25), we obtain

$$S_{B}=\frac{1}{x_{B}}\frac{\Gamma_{B}+{\overset{-}{\rho }}_{B}H}{\Gamma_{A}+\Gamma_{B}+({\overset{-}{\rho }}_{A}+{\overset{-}{\rho }}_{B})H}$$ 28

This exact relation makes explicit that the adsorptions of species A and B are the key thermodynamic quantities governing the pore selectivity. Note that *S*_B_ is independent of the definition of *H*. Given the flexibility afforded by this invariance, we choose *H* such that Γ_B_ = 0 when *S*_B_ = 1; this is only possible if Γ_A_ also vanishes. That is, the relative adsorption,

$$\Gamma_{A}^{\left(B\right)}=\Gamma_{A}-({\overset{-}{\rho }}_{A}/{\overset{-}{\rho }}_{B})\Gamma_{B}$$ 29

is zero at *S*_B_ = 1. This result is confirmed in Figure 4a and Figure 4b, where we show how *S*_B_ and Γ_A_^(B)^, respectively, vary with *x*_B_ at various different thermodynamic state points, for a slit pore with βϵ_w,A_ = βϵ_w,B_ = 2.0 and *L* = 8σ.

**Figure 4:**
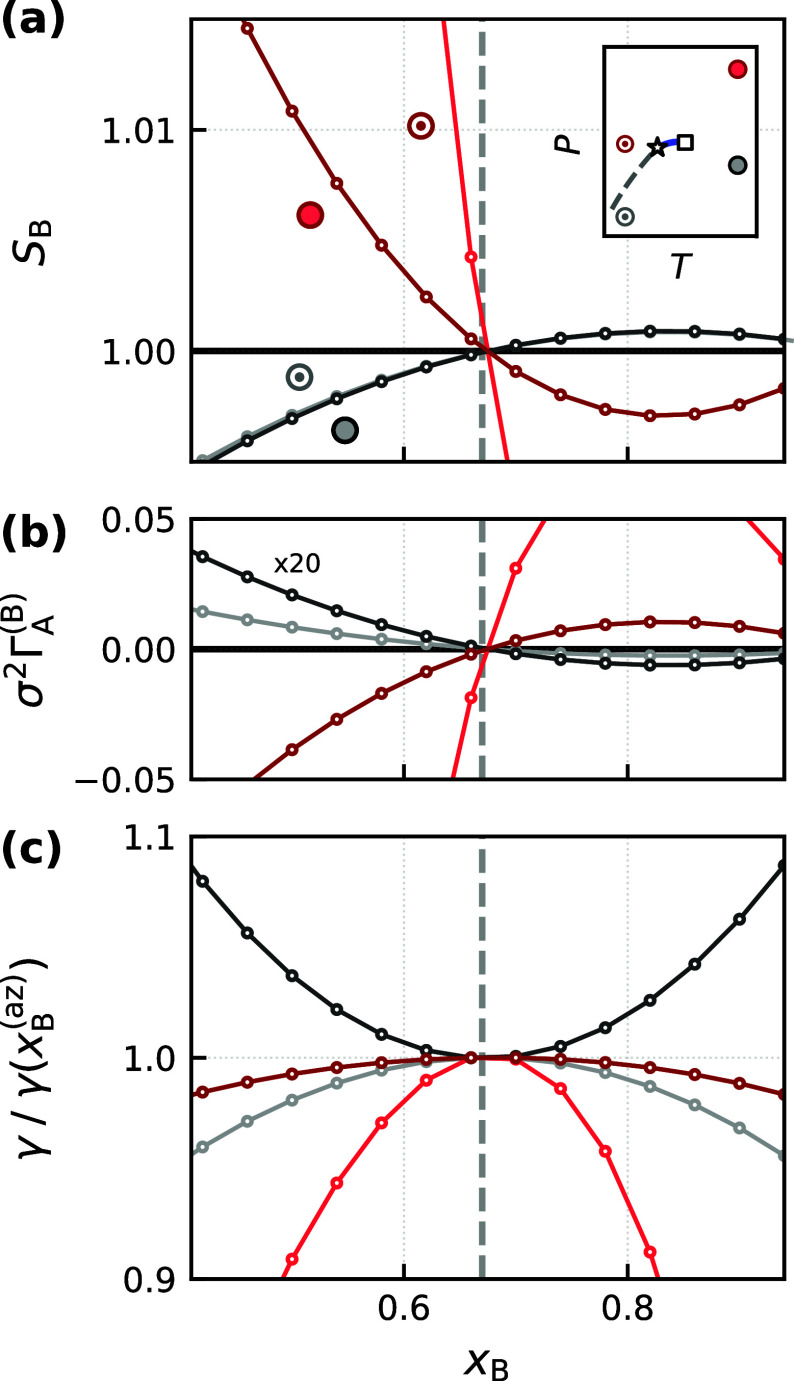
Coincidence of *S*_B_ = 1, vanishing relative adsorption, and extremal interfacial tension. (a) *S*_B_ vs *x*_B_ at the four different state points from Figure 2 (and indicated in the inset). (b) Γ_A_^(B)^ vs *x*_B_, with results corresponding to low pressures (light and dark gray) multiplied by a factor of 20 for clarity. (c) Wall–fluid interfacial tension. The vertical dashed line indicates the azeotropic composition. All results correspond to a slit pore with βϵ_w,A_ = βϵ_w,B_ = 2.0 and *L* = 8σ.

The point at which the relative adsorption is zero defines the aneotropic composition.^91–93^ For the liquid–vapor interface of a similar binary LJ mixture to that studied here, Telo da Gama and Evans^92^ also reported that *x*_B_^(an)^ ≈ *x*_B_^(az)^, for which they provided a qualitative explanation: at low *x*_B_, the vapor phase is relatively enriched in species B, leading to preferential adsorption of B; at *x*_B_ = *x*_B_^(az)^, the liquid and vapor phases have identical compositions and the adsorption vanishes; for high *x*_B_ the liquid becomes relatively richer in species B than the vapor, causing the adsorption to change sign. In other words, the sign of Γ_A_^(B)^ is controlled by whether the bulk vapor or bulk liquid phase is enriched in species B. Based on our results, it would seem that, when the walls interact with A and B in the same manner, a similar qualitative understanding extends to fluids under confinement.

Telo da Gama and Evans also found that the liquid–vapor surface tension is minimum at the aneotropic point. It is natural, then, to explore the extent to which the aneotropic point influences the wall–fluid interfacial tension in these confined systems. From eq 27, and using the Gibbs–Duhem relation, we find

$${\left(\frac{\partial \gamma }{\partial x_{B}}\right)}_{H,P,T}=-\frac{1}{2}{\left(\frac{\partial \mu_{A}}{\partial x_{B}}\right)}_{P,T}\left[\Gamma_{A}-\left(\frac{1-x_{B}}{x_{B}}\right)\Gamma_{B}\right]$$ 30

Evaluating at *x*_B_ = *x*_B_^(an)^, where Γ_A_ = Γ_B_ = 0, we have

$${\left(\frac{\partial \gamma }{\partial x_{B}}\right)}_{H,P,T}{|}_{x_{B}=x_{B}^{\left(\mathrm{an}\right)}}=0$$ 31

While this result is consistent with the minimum of the liquid–vapor surface tension reported in ref 92, more generally, it states that the aneotropic point corresponds to an extremum in wall–fluid interfacial tension. This prediction is borne out by results from neural LMFT; as seen in Figure 4c, γ­(*x* ^(an)^) changes from a local minimum to a local maximum as the system traverses from high to low pressures.

The above arguments readily extend to cases where the relative wall affinity Δϵ_w_ = ϵ_w,B_ – ϵ_w,A_ ≠ 0. Intuitively, we expect that varying Δϵ_w_ will change the pore selectivity, and the aneotropic point. This notion is confirmed in Figure 5a–c, where we respectively show how *S*_B_, Γ_A_^(B)^, and γ vary with *x*_B_, with βΔϵ_w_ = ±0.2, for a system at *k*_B_ *T*/ϵ = 0.77, *P*σ^3^/ϵ = 0.0248, and *L* = 8σ. As expected, we see that *S*_B_ = 1 coincides with the aneotropic composition. The results also appear consistent with a minimum in interfacial tension, albeit a shallow one. Due to this shallowness, we have performed fits to a fourth-order polynomial, constrained to be minimum at *x*_B_^(an)^, as shown by the solid black lines in Figure 5c.

**Figure 5:**
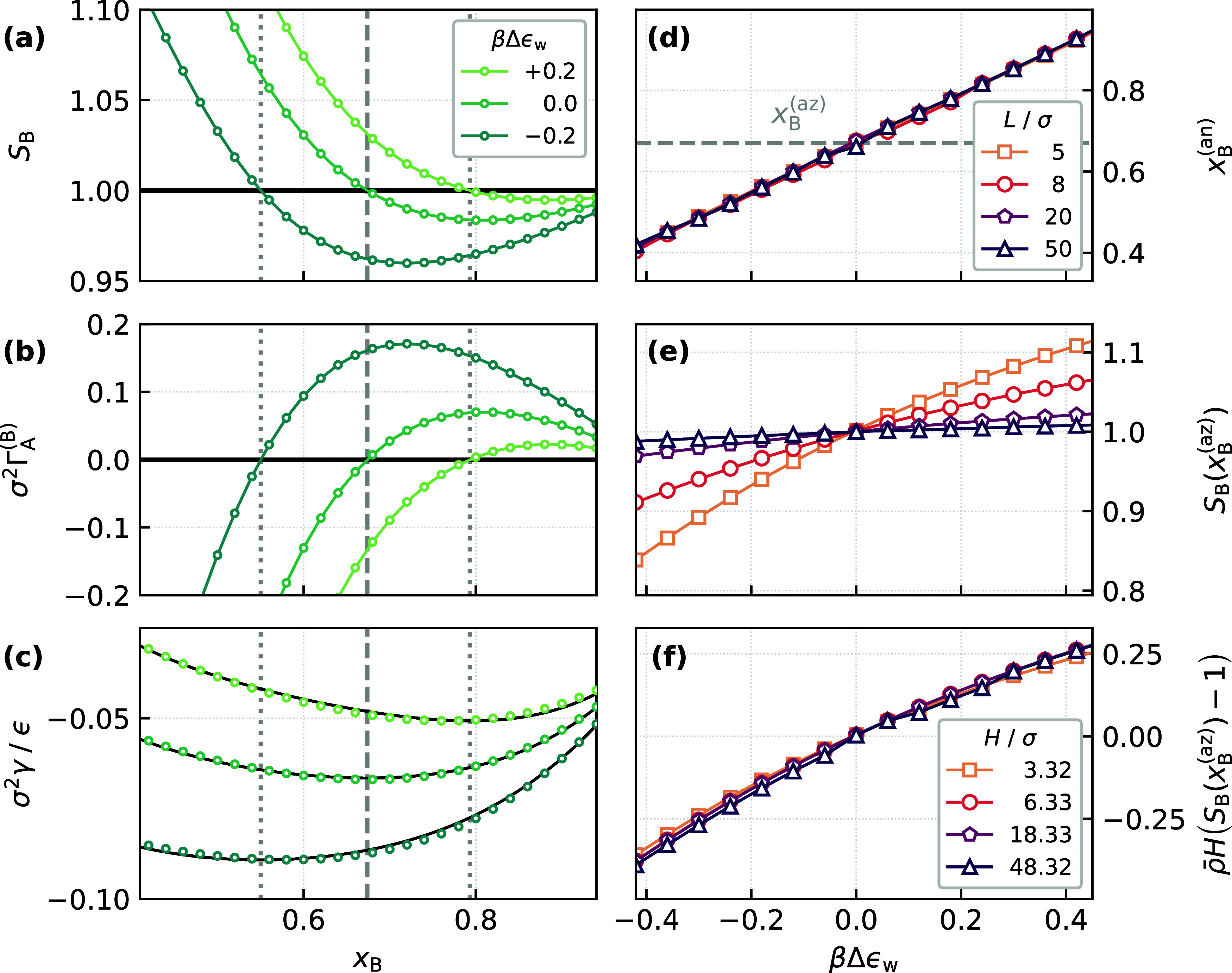
Effect of wall affinity, Δϵ_w_ = ϵ_w,B_ – ϵ_w,A_, on the pore selectivity. In all cases, βϵ_w,B_ = 2.0. (a), (b), and (c) respectively show how *S*_B_, Γ_A_^(B)^, and γ vary with *x*_B_. In all cases, we observe that *S*_B_ = 1, Γ_A_^(B)^ = 0, and a minimum in γ coincide. The vertical dashed and dotted lines indicate *x*_B_^(az)^ and *x*_B_^(an)^, respectively. The solid black lines in (c) show fits to a fourth-order polynomial, constrained to be minimum at the aneotropic composition. Panel (d) shows how *x*_B_^(an)^ varies with βΔϵ_w_ for different *L* (as indicated in the legend). (e) *S*_B_(*x*_B_^(az)^) vs βΔϵ_w_ for different widths of the slit pore. When rescaled according to eq 32 all data approximately collapse onto the same master curve, as seen in (f).

In Figure 5d, we show how *x* ^(an)^ varies with βϵ_w_ for different slit widths. To a very good approximation, the variation is linear, and insensitive to *L*. We interrogate this size-independence further by considering how selectivity varies with Δϵ_w_ within the context of the thermodynamic model presented above. As we have established that *x*_B_^(an)^ ≈ *x*_B_^(az)^ when Δϵ_w_ = 0, it is a straightforward, though slightly tedious, matter to show that

$$\overset{-}{\rho }H(S_{B}(x_{B}^{\left(\mathrm{az}\right)};\;\Delta \epsilon_{w})-1)=-\Delta \epsilon_{w}{\left(\frac{\partial \Gamma_{A}^{\left(B\right)}(x_{B}^{\left(\mathrm{az}\right)};\;0)}{\partial \Delta \epsilon_{w}}\right)}_{H,P,T}$$ 32

In Figure 5e we show how *S*_B_(*x*_B_^(az)^) varies with Δϵ_w_, where we see that the variation is nonlinear, which becomes more pronounced for smaller slit pores. In Figure 5f, we plot the left-hand side of eq 32 vs Δϵ_w_ for different slit widths. Strikingly, we observe that all data approximately collapse onto the same curve. This insensitivity to the slit width suggests that, even for systems barely large enough to accommodate three layers of particles, the two interfaces of the slit pore essentially behave independently; in this case, down to the range of the wall–fluid interaction.

## 4 Conclusions

In this study, we have extended the recently developed neural density functional theory to a binary mixture of Lennard–Jones particles that exhibits azeotropic phase behavior. In contrast to another recent neural functional theory study on mixtures,^21^ we have used machine learning to obtain an accurate representation of a *single-component* repulsive reference system, and treated attractive interactions in a mean-field fashion. The mean-field approach that we adopt is rooted in the connection between classical density functional theory (and its recent extension, hyperdensity functional theory), and local molecular field theory derived by Weeks and co-workers.^73–75^ Within this neural LMFT framework, we have taken advantage of the fact that, when known from other sources,^70,71^ the bulk equation of state of the fluid can be integrated seamlessly, allowing us to focus on applying density functional theory itself to inhomogeneous systems.

We have used this neural LMFT framework to understand preferential adsorption of this binary fluid in a slit-pore geometry. In cases where the walls of the slit pore interact with both species of the fluid in the same manner, our numerical results indicate that when the reservoir is at its azeotropic composition, so too is the composition in the pore. Remarkably, this observation persists across a broad range of thermodynamic conditions, including far into the supercritical state. By analyzing the bulk equation of state, we find that the azeotropic composition coincides with equal partial molar volumes of the constituent species, and a local extremum of the isothermal compressibility. Intriguing as these observations are, their generality to other fluids that exhibit azeotropy is an open question that warrants further investigation. For example, the system we have investigated has an azeotropic composition that is largely insensitive to changes in temperature and pressure; this does not generally hold, especially when the components have markedly different sizes.^102^

To elucidate the mechanisms underlying pore selectivity, we have presented a thermodynamic description that connects selectivity directly to interfacial adsorption. This shows that the aneotropic composition, defined by vanishing relative adsorption, remains closely tied to the bulk azeotropic composition over a wide range of thermodynamic conditions, even under confinement. We show clearly that the relative adsorption of each species in the mixture is the relevant thermodynamic driving force, with the wall–fluid interfacial tension reaching an extremum at the aneotropic composition—a result that generalizes previous work on the liquid–vapor interface^92^ to confined systems. By analyzing our numerical results within the context of this thermodynamic model, we found that the aneotropic composition shifts linearly with the relative affinity of the confining walls to the two species. Moreover, we found that the two interfaces of the slit pore act essentially independently down to remarkably small separations—a little over three molecular diameters—between the walls.

With machine learning techniques, it is now possible to accurately model systems of remarkable complexity—both in terms of their interactions and emergent phase behavior—using classical density functional theory. Here, we take “classical density functional theory” in a broad sense to encompass its recent extensions hyper-DFT^57^ and meta-DFT.^103^ We have used the connections between local molecular field theory and cDFT as a means to justify a mean-field treatment of attractive interactions, as well as to incorporate an established bulk equation of state into the framework. By learning the one-body direct correlation function once for a single-component system, which we then used as a reference for a binary fluid, we have demonstrated a “train once, learn many” strategy as a proof-of-principle. For more complex systems, especially mixtures where particle sizes differ significantly, it may prove fruitful to combine this strategy with meta-DFT, which aims to learn the functional dependence on the interaction potential directly. Other developments in applying ML to obtain bulk equations of state may also prove useful.^104,105^ Irrespective of the exact strategy that one adopts, it seems highly likely that classical density functional theory combined with machine learning will play an increasingly important role in understanding fluids relevant to physical chemistry.

## Supplementary Material



## Data Availability

Data and code supporting the findings of this study are openly available at Zenodo (https://doi.org/10.5281/zenodo.19250207) and Github (https://github.com/CoxGroup/lj-cdft-lmft).

## Notes

Additional results validating the accuracy of the mean-field neural LMFT approach, comparison to standard mean-field cDFT, and additional calculations for other binary LJ mixtures (PDF)
