# Supplementary material for: Roles of Bulk and Surface Thermodynamics in the Selective Adsorption of a Confined Azeotropic Mixture
Source: J Phys Chem B. 2026 Apr 9;130(16):4455–66. doi: 10.1021/acs.jpcb.6c00640 (PMC13112351; doi:10.1021/acs.jpcb.6c00640)
Supplement: Supplementary file 1 [file jp6c00640_si_001.pdf]

Supplementary Information: Roles of Bulk and Surface Thermodynamics in the Selective Adsorption of a Confined Azeotropic Mixture

Katie L. Y. Zhou,[1] Anna T. Bui,[2,1] and Stephen J. Cox[1,a)]
[1)] *Department of Chemistry, Durham University, South Road, Durham, DH1 3LE, United Kingdom*
[2)] *Yusuf Hamied Department of Chemistry, University of Cambridge, Lensfield Road, Cambridge, CB2 1EW, United Kingdom*

(Dated: March 30, 2026)

S1. COMPARISON TO SIMULATION AND STANDARD MEAN-FIELD THEORY

Archer and Evans previously established a connection between LMFT and traditional mean-field cDFT.[1] While similar to our formalism, $\Delta\mu$ was identified as an integration constant, which for mixtures reads

$$\Delta\mu_\alpha = \sum_\eta \bar{\rho}_\eta \int \mathrm{d}\boldsymbol{r}'\, u_{1,\alpha\eta}(|\boldsymbol{r}'|)\,. \tag{S1}$$

In this section, we compare the neural LMFT approach to the standard mean-field cDFT treatment. That is, we consider the above expression (Eq. S1) for $\{\Delta\mu_\alpha\}$ together with a hard sphere (HS) reference; here we use the accurate neural functional from Ref. 2.

In Fig. S1 we show a comparison between the neural LMFT framework and standard mean-field cDFT for capillary condensation (Fig. 1b in the main text), with a reservoir at $k_\mathrm{B}T/\epsilon = 0.77$, $P\sigma^3/\epsilon = 0.020$, and $x_\mathrm{B} = 0.78$, which corresponds to the vapor state. Both approaches show the transition from a gas-like to liquid-like state when going from interaction strength $\epsilon_\mathrm{w,A} = \epsilon_\mathrm{w,B} = 2.0k_\mathrm{B}T$ to $2.5k_\mathrm{B}T$. While both are in excellent agreement with GCMC simulation for the gas-like state, they begin to deviate for the higher-density liquid-like state. Minor discrepancies are observed with neural LMFT, while the standard mean-field result overestimates the peaks in the density profiles.

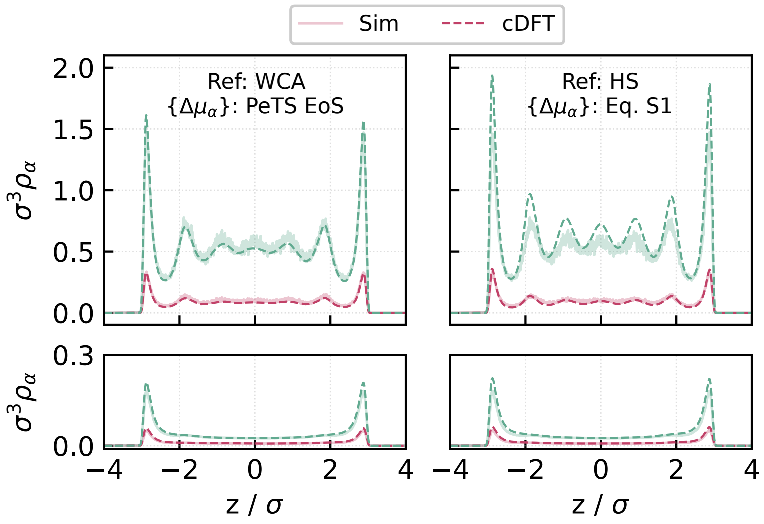

Figure S1: Comparison with the standard mean-field cDFT treatment for capillary condensation. Left: neural LMFT, using a WCA reference and $\{\Delta\mu_\alpha\}$ from the PeTS equation of state. Right: standard mean-field cDFT, using a HS reference and Eq. S1 for $\{\Delta\mu_\alpha\}$. Top row shows the condensed liquid state with $\epsilon_\mathrm{w,A} = \epsilon_\mathrm{w,B} = 2.5\,k_\mathrm{B}T$, while the bottom row shows the vapor state at $\epsilon_\mathrm{w,A} = \epsilon_\mathrm{w,B} = 2.0\,k_\mathrm{B}T$.

[a)] Electronic mail: stephen.j.cox@durham.ac.uk

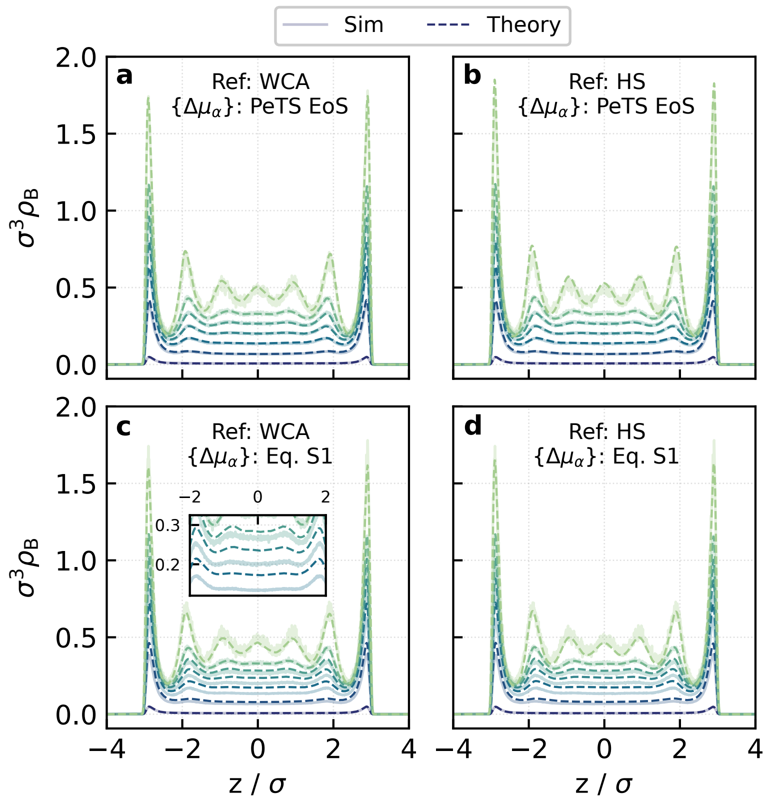

Figure S2: Comparison of reference systems and cDFT approaches at different bulk densities at supercritical conditions. (a) Neural LMFT, using the WCA reference and the PeTS equation of state. (b) HS reference and the PeTS equation of state. (c) WCA reference and $\{\Delta\mu_\alpha\}$ given by Eq. S1. (d) Standard mean-field cDFT, using a HS reference and $\{\Delta\mu_\alpha\}$ given by Eq. S1.

In Fig. S2 we investigate the factors governing these differences in more detail, such as the reference system and the treatment of $\{\Delta\mu_\alpha\}$. The reservoir is now in a supercritical state at $k_\mathrm{B}T/\epsilon = 1.50$ and $x_\mathrm{B} = 0.66$, with the total density varied between $\sigma^3\bar{\rho} = 0.01$ to $\sigma^3\bar{\rho} = 0.7$. The two walls are separated by $L = 8\sigma$ and interact with strength $\epsilon_{\mathrm{w,A}} = \epsilon_{\mathrm{w,B}} = 2.0\,k_\mathrm{B}T$. As observed in Fig. S2a, neural LMFT, i.e., using the WCA reference and the PeTS equation of state for $\{\Delta\mu_\alpha\}$, results in excellent agreement with GCMC simulation across all densities. Switching to a HS reference in Fig. S2b results in similarly excellent agreement for most densities, though the system is over-structured at $\sigma^3\bar{\rho} = 0.7$. In Figs. S2c and S2d, we respectively use WCA and HS as reference, but $\{\Delta\mu_\alpha\}$ is obtained with Eq. S1. While both reference systems show excellent agreement at the highest and lowest densities, neither obtains the correct bulk density at intermediate densities. This is in line with observations made with LMFT.[3,4]

Highlighting further the differences in bulk thermodynamics used by mean-field theory and the two reference systems, we present the binodal in Fig. S3 for the single-component truncated and shifted LJ fluid. The neural LMFT approach, by construction, reproduces the same outputs as that from the PeTS equation of state. Fig. S3a shows an isotherm of the chemical potential as a function of bulk density at $k_\mathrm{B}T/\epsilon = 0.90$. All approaches show a van der Waals loop. Performing a Maxwell construction on these isotherms gives the binodal in Fig. S3b. Comparing to simulation results from Ref. 5, the PeTS equation of state (and by extension, neural LMFT) is the most accurate. The mean-field theories can accurately reproduce the vapor coexistence densities, though the WCA reference underestimates the liquid densities. The HS reference, through cancellation of errors, results in accurate liquid densities below the critical temperature.

In addition to the errors with the coexistence densities, the mean-field approaches overestimate the critical temperature and underestimate the critical density. Near the critical temperature, an empirical fit can be made of the form[6,7]

$$\rho_\pm = a|T - T_\mathrm{c}| \pm b|T - T_\mathrm{c}|^\beta + \rho_\mathrm{c} \tag{S2}$$

where $\rho_+ = \rho^{(\mathrm{l})}$ and $\rho_- = \rho^{(\mathrm{v})}$ are the liquid and vapor coexistence densities respectively, ρ_c is the critical

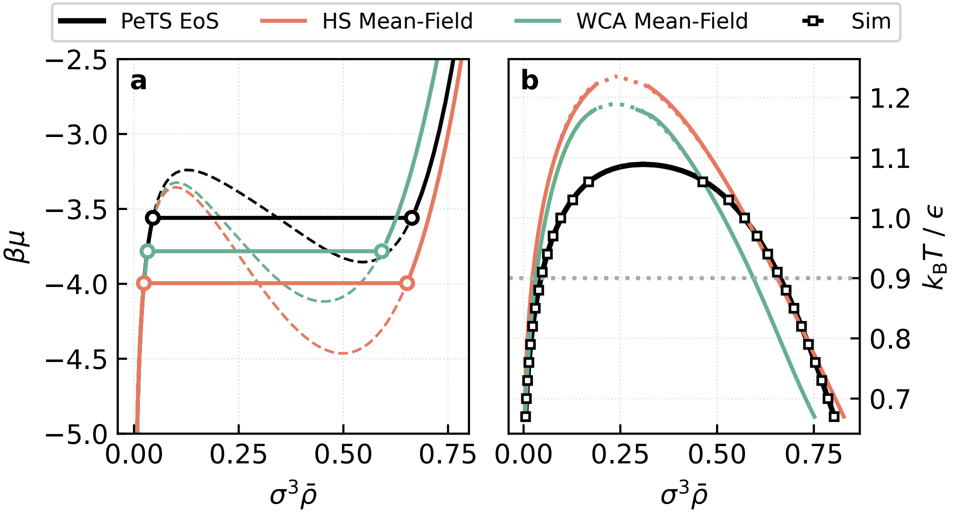

Figure S3: Bulk liquid–vapor coexistence for the single-component LJ fluid, comparing the EoS against the standard mean-field treatment with different reference systems. (a) The chemical potential as a function of bulk density at $k_BT/\epsilon = 0.90$, highlighted by the dotted grey line on the right. The coexistence densities obtained from Maxwell construction are shown by the circles. (b) The binodal obtained from Maxwell construction. Fits of the binodal to Eq. S2 are shown by the colored dotted lines. Simulation results are from Vrabec *et al.* in Ref. 5.

Table S1: Critical exponents extracted from the fits to the binodals (Eq. S2). The number in parentheses indicates the uncertainty in the last digit.

	HS Mean-Field	WCA Mean-Field	PeTS	Literature
k_BT_c/ϵ	1.236(1)	1.1909(6)	1.089[9]	1.0779[5]
$\sigma^3\rho_c$	0.2422(7)	0.2381(4)	0.3092[9]	0.3190[5]
β	0.500(3)	0.472(2)	0.350(2)	0.32630(22)[8]

density, β is the standard 3D Ising result,[8] and a and b are parameters to be fitted. Results are presented in Table S1. The PeTS equation of state returns values that are closest to those observed in literature.

S2. RESULTS FOR OTHER SYSTEMS

One of the main benefits of using a single-component reference system is that it lends itself readily to a "train once, learn many" strategy. As further demonstration of this transferability, we show brief results in Fig. S4. Density profiles of truncated and shifted LJ binary mixtures with varying ϵ_{BB} and ϵ_{AB} are presented in Fig. S4a and compared to GCMC simulation. Parameters of the mixtures are summarized in Table S2. The bulk reservoir is at a supercritical state with $k_{\mathrm{B}}T/\epsilon = 1.50$, with $\beta\mu_{\mathrm{A}} = \beta\mu_{\mathrm{B}} = -2.0$. The two walls interact with strength $\epsilon_{\mathrm{w,A}} = \epsilon_{\mathrm{w,B}} = 2.0\,k_{\mathrm{B}}T$. Excellent agreement between neural LMFT and the simulation results can be seen. The same pore selectivity analysis conducted in the main paper can easily be extended to these other mixtures. In Fig. S4b, results are presented for the position of the aneotrope with $\epsilon_{\mathrm{w,B}} = 2.0\,k_{\mathrm{B}}T$ and varying $\Delta\epsilon_{\mathrm{w}}$ for systems confined in a slit of width $L = 8\sigma$. The bulk reservoir is at $k_{\mathrm{B}}T/\epsilon = 0.77$ and $P\sigma^3/\epsilon = 0.10$, a bulk liquid for all systems. The interaction between unlike species is varied whilst ϵ_{BB} is kept at 0.9ϵ. Like with the system studied in the main paper, we find that for the case of symmetric walls $x_{\mathrm{B}}^{(\mathrm{an})} \approx x_{\mathrm{B}}^{(\mathrm{az})}$. Varying the asymmetry of the wall potential leads to shifts in $x_{\mathrm{B}}^{(\mathrm{an})}$.

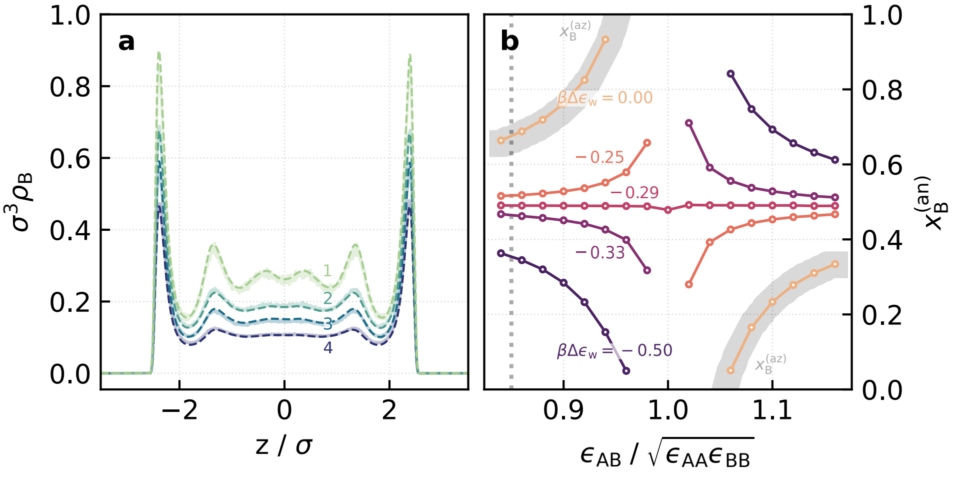

Figure S4: Varying ϵ_{BB} and ϵ_{AB} of binary LJ mixtures. (a) Density profiles of different binary LJ systems; the parameters are summarized in Table S2. Excellent agreement to GCMC simulations can be seen. (b) The position of the aneotrope ($S_{\mathrm{B}} = 1$) calculated for varying $\Delta\epsilon_{\mathrm{w}}$ and ϵ_{AB} for systems with $\epsilon_{\mathrm{BB}} = 0.9\epsilon$. The shaded grey regions indicate the azeotropic composition, obtained from the PeTS equation of state.[10,11] The dotted line indicates the system studied in the main paper i.e., $\epsilon_{\mathrm{AB}} = 0.806\epsilon$.

Table S2: Parameters of the LJ binary mixtures in Fig. S4a. Mixture 3 is the same as that studied in the main paper.

Mixture	$\epsilon_{\mathrm{BB}}/\epsilon_{\mathrm{AA}}$	$\epsilon_{\mathrm{AB}}/\sqrt{\epsilon_{\mathrm{AA}}\epsilon_{\mathrm{BB}}}$
1	0.5	0.85
2	0.5	1.25
3	0.9	0.85
4	0.9	1.25

REFERENCES

[1] A. J. Archer and R. Evans, "Relationship between local molecular field theory and density functional theory for non-uniform liquids," J. Chem. Phys. **138**, 014502 (2013).

[2] F. Sammüller, S. Hermann, D. De Las Heras, and M. Schmidt, "Neural functional theory for inhomogeneous fluids: Fundamentals and applications," Proc. Natl. Acad. Sci. **120**, e2312484120 (2023).

[3] J. D. Weeks, K. Vollmayr, and K. Katsov, "Intermolecular forces and the structure of uniform and nonuniform fluids," Phys. Stat. Mech. Its Appl. **244**, 461–475 (1997).

[4] J. D. Weeks, K. Katsov, and K. Vollmayr, "Roles of Repulsive and Attractive Forces in Determining the Structure of Nonuniform Liquids: Generalized Mean Field Theory," Phys. Rev. Lett. **81**, 4400–4403 (1998).

[5] J. Vrabec, G. K. Kedia, G. Fuchs, and H. Hasse, "Comprehensive study of the vapour–liquid coexistence of the truncated and shifted Lennard–Jones fluid including planar and spherical interface properties," Mol. Phys. **104**, 1509–1527 (2006).

[6] F. Sammüller, M. Schmidt, and R. Evans, "Neural Density Functional Theory of Liquid-Gas Phase Coexistence," Phys. Rev. X **15**, 011013 (2025).

[7] N. B. Wilding, "Critical-point and coexistence-curve properties of the Lennard-Jones fluid: A finite-size scaling study," Phys. Rev. E **52**, 602–611 (1995).

[8] A. M. Ferrenberg, J. Xu, and D. P. Landau, "Pushing the limits of Monte Carlo simulations for the three-dimensional Ising model," Phys. Rev. E **97**, 043301 (2018).

[9] S. Stephan, J. Liu, K. Langenbach, W. G. Chapman, and H. Hasse, "Vapor-Liquid Interface of the Lennard-Jones Truncated and Shifted Fluid: Comparison of Molecular Simulation, Density Gradient Theory, and Density Functional Theory," J. Phys. Chem. C **122**, 24705–24715 (2018).

[10] M. Heier, S. Stephan, J. Liu, W. G. Chapman, H. Hasse, and K. Langenbach, "Equation of state for the Lennard-Jones truncated and shifted fluid with a cut-off radius of 2.5 σ based on perturbation theory and its applications to interfacial thermodynamics," Mol. Phys. **116**, 2083–2094 (2018).

[11] P. Rehner, G. Bauer, and J. Gross, "FeOs: An Open-Source Framework for Equations of State and Classical Density Functional Theory," Ind. Eng. Chem. Res. **62**, 5347–5357 (2023).
